# Potential mechanisms underlying podophyllotoxin-induced cardiotoxicity in male rats: toxicological evidence chain (TEC) concept

**DOI:** 10.3389/fphar.2024.1378758

**Published:** 2024-09-24

**Authors:** Kaiyue Ma, Lu Sun, Chunxue Jia, Hongqian Kui, Jiaqi Xie, Shidan Zang, Shixin Huang, Jinfeng Que, Chuanxin Liu, Jianmei Huang

**Affiliations:** ^1^ School of Chinese Materia Medica, Beijing University of Chinese Medicine, Beijing, China; ^2^ College of Chinese Materia Medica and Food Engineering, Shanxi University of Chinese Medicine, Jinzhong, China; ^3^ Eye Hospital China Academy of Chinese Medical Sciences, Beijing, China; ^4^ Luoyang Key Laboratory of Clinical Multiomics and Translational Medicine, Henan Key Laboratory of Rare Diseases, Endocrinology and Metabolism Center, The First Affiliated Hospital, and College of Clinical Medicine of Henan University of Science and Technology, Luoyang, China

**Keywords:** toxicological evidence chain (TEC), podophyllotoxin, cardiotoxicity, transcriptomics, arachidonic acid metabolism

## Abstract

**Introduction:**

Podophyllotoxin (PPT) is a high-content and high-activity compound extracted from the traditional Chinese medicinal plant *Dysosma versipellis* (*DV*) which exhibits various biological activities. However, its severe toxicity limits its use. In clinical settings, patients with *DV* poisoning often experience adverse reactions when taking large doses in a short period. The heart is an important toxic target organ, so it is necessary to conduct 24-h acute cardiac toxicity studies on PPT to understand its underlying toxicity mechanism.

**Methods:**

Based on the concept of the toxicological evidence chain (TEC), we utilized targeted metabolomic and transcriptomic analyses to reveal the mechanism of the acute cardiotoxicity of PPT. The manifestation of toxicity in Sprague-Dawley rats, including changes in weight and behavior, served as Injury Phenotype Evidence (IPE). To determine Adverse Outcomes Evidence (AOE), the hearts of the rats were evaluated through histopathological examination and by measuring myocardial enzyme and cardiac injury markers levels. Additionally, transcriptome analysis, metabolome analysis, myocardial enzymes, and cardiac injury markers were integrated to obtain Toxic Event Evidence (TEE) using correlation analysis.

**Results:**

The experiment showed significant epistaxis, hypokinesia, and hunched posture in PPT group rats within 24 h after exposure to 120 mg/kg PPT. It is found that PPT induced cardiac injury in rats within 24 h, as evidenced by increased serum myocardial enzyme levels, elevated concentrations of cardiac injury biomarkers, and altered cardiac cell morphology, all indicating some degree of cardiac toxicity. Transcriptome analysis revealed that primary altered metabolic pathway was arachidonic acid metabolism after PPT exposure. Cyp2e1, Aldob were positively correlated with differential metabolites, while DHA showed positive correlation with differential genes Fmo2 and Timd2, as well as with heart injury markers BNP and Mb.

**Conclusion:**

This study comprehensively evaluated cardiac toxicity of PPT and initially revealed the mechanism of PPT-induced acute cardiotoxicity, which involved oxidative stress, apoptosis, inflammatory response, and energy metabolism disorder.

## 1 Introduction

Podophyllotoxin (PPT) is a high-content and high-activity compound extracted from a plant in traditional Chinese medicine (TCM), *Dysosma versipellis* (*DV*). Its content in the *DV* raw drug can reach up to 9%. PPT exhibits various biological activities, such as anti-tumor, antiviral, and anti-inflammatory effects. In particular, its anti-tumor activity is evidenced by the inhibition of the proliferation of non-small-cell lung cancer, human oral squamous cell carcinoma, and other cancer cells (Pathak et al., 2022; Tan et al., 2018; Zhao et al., 2021). However, the toxicity and serious gastrointestinal adverse reactions of PPT, as well as the lack of specific drugs after poisoning, limit its clinical application (Kong et al., 2023). The toxicity caused by *DV* involves multiple organs, with the heart as an important target. Thus, PPT may cause damage to the heart, but this has not been confirmed, and the related toxicity mechanisms are not yet clear. Therefore, there is a need for a systematic and comprehensive analysis to explore the cardiotoxicity and mechanism of PPT action to facilitate its treatment utility.

The toxicological evidence chain (TEC) framework was first proposed and applied to research potential toxic material bases and mechanisms of compounds and natural drugs in 2019 by our research group (Liu et al., 2020). The current TEC framework comprises four core contents. Injury phenotype evidence (IPE) involves examining the overall animal level behavioral status after administering TCM in model animals and qualitatively and quantitatively characterizing the toxic behavior and toxic manifestations of animals. Harmful ingredients evidence (HIE) examines the drug-derived component or substance basis related to toxic outcomes. Toxic events evidence (TEE) involves obtaining endogenous differences in key targets, metabolic small molecules, and signal transduction in the process of toxicity. Adverse outcomes evidence (AOE) involves investigating the substantial detriment to target organs (Kong et al., 2022; Duan et al., 2024). This study was based on the TEC method, where we constructed a TEC framework for PPT and demonstrated the dynamic relationship between damage events and toxicity effects. The combined application of high-throughput omics technology provides guidance for comprehensive and multi-angle toxicity assessment. Transcriptomics uses high-throughput sequencing technology to compare normal and exposed individuals, identify different messenger ribonucleic acids related to toxicity, and discover potential genomic biomarkers of PPT cardiotoxicity. Targeted metabolomics uses highly sensitive detection methods to quantitatively analyze specific endogenous small-molecule compounds and to screen potential biomarkers related to cardiotoxicity.

In this study, we first determined the changes in epimorphology, serum biochemical indicators, cardiac injury markers, and cardiac tissue morphology in Sprague–Dawley (SD) male rats exposed to PPT. Subsequently, transcriptome characteristics and arachidonic acid-targeted metabolomic profiles were revealed, utilizing heart transcriptomics and targeted metabolomics approaches. Finally, transcriptome analysis, metabolome analysis, myocardial enzymes, and cardiac injury markers were integrated to explore the cardiotoxic mechanism of PPT.

## 2 Materials and methods

### 2.1 Reagents and materials

PPT (≥ 98%, 518-28-5) was purchased from Shanghai YUANYE Biotechnology Co., Ltd. In animal experiments, pentobarbital (Pentobarbital Sodium, P3761, Sigma, United States) was used to induce anesthesia, and sodium chloride (AR, S24119, Shanghai YUANYE Biotechnology Co., Ltd.) and 4% paraformaldehyde (R20486, 500 mL, Shanghai YUANYE Biotechnology Co., Ltd.) were used to wash and fix the hearts of the rats. ELISA kits for rat myoglobin (MYO/MB), rat brain natriuretic peptide/brain natriuretic peptide (BNP), rat cardiac troponin I (cTnI), and rat N-terminal pro-brain natriuretic peptide (NT-proBNP) were purchased from Jiangsu Meimian Industrial Co. Ltd. (batch numbers 2023082415R, 2023082467, 2023082450R, and 2023082429R, respectively). The mobile phases were acetonitrile (LC-MS, 192552, Thermo Fisher, United States), formic acid (LC-MS, 225583, Thermo Fisher, United States), methanol (LC-MS, 190379, Thermo Fisher, United States), and ultra-pure water from the Milli-Q Integral water purification system (Millipore, United States).

### 2.2 Toxicological research on cardiotoxicity induced by PPT (IPE and AOE)

#### 2.2.1 Animal handling

Forty specific-pathogen-free (SPF) male SD rats weighing 230 ± 10 g and aged 7–8 weeks were purchased from Beijing Vital River Laboratory Animal Technology Co., Ltd., license number SCXK (Beijing-2021-0006), and raised in the Animal Laboratory of Liangxiang Campus, Beijing University of Chinese Medicine. The rats were fed under controlled environmental conditions with a 12-h day–night cycle, an ambient temperature of 23°C ± 2°C, and an ambient humidity of 35% ± 5%, with adaptive feeding for 3 days. Rats were randomly divided into two groups: blank control group (CON) and podophyllotoxin-treated group (PPT). The dosage of each group is shown in Table 1. After administration, pentobarbital sodium was injected to anesthetize the rats, and their hearts were removed. Animal experiment protocols were approved by the Animal Ethics Committee of Beijing University of Chinese Medicine (BUCM-2023042701-2049). All experimental procedures were conducted following the Chinese national legislation and local guidelines.

**TABLE 1:** Administration scheme of cardiotoxicity induced by PPT in rats.

Group	Number	Drug	Dose	Administration method	Exposure period (d)
CON	20	Ultra-pure water	1 mL/d	i.g., Single-dose administration	1
PPT	20	PPT solution	120 mg/kg		1

#### 2.2.2 Drug preparation

Drug solutions with a concentration of 100 mg/mL were prepared and diluted to the dose required for administration. In brief, we accurately weighed the appropriate amount of PPT, dissolved it in ultra-pure water as required, stored it in a refrigerator at 4 °C, and thawed it at room temperature before use. The solution for the blank control group was ultra-pure water. To ensure uniformity of gavage, we administered PPT to rats one by one and vortexed the PPT solution for 30 s to 1 min each time to ensure a uniform suspension.

#### 2.2.3 Acute cardiac injury phenotype induced by PPT

After the CON and PPT groups were administered the drugs/blank, the rats’ general behavior, appearance changes, general morphological changes, and deaths were observed, including changes in eye color, paws, and hair, bleeding, and the occurrence of diarrhea (Supplementary Table S1). Body weight changes were recorded every hour 0–4 h after administration, every 2 h 5–12 h after administration, and every 12 h 13–24 h after administration (GB/T 21757-2008, 2008). After administering the corresponding medication by gavage to the corresponding group of rats for 24 h, blood was drawn from the abdominal aorta. After blood collection, physiological saline was perfused from the liver at 0 °C without touching the heart until the lungs turned white. The rat heart was immediately collected and weighed to calculate its organ indexes.

#### 2.2.4 Detection of myocardial enzyme and cardiac injury markers

The whole blood (8–10 mL) in a general vacuum tube was centrifuged once at 3,500 rpm, 4 °C for 15 min. The supernatant was stored in a refrigerator at −80 °C until myocardial enzymes biochemical parameters related to cardiac function were detected, including aspartate aminotransferase (AST), creatine kinase (CK), creatine kinase-MB isoenzyme (CK-MB), lactate dehydrogenase (LDH), and α-hydroxybutyrate dehydrogenase (α-HBD), as well as cardiac injury markers including brain natriuretic peptide/brain natriuretic peptide (BNP), cardiac troponin I (cTnI), myoglobin (MYO/MB), and N-terminal pro-brain natriuretic peptide (NT-proBNP). Mb, cTnI, BNP, and NT pro-BNP in blood were quantified by the ELISA kit following the manufacturer’s instructions, and the optical density of each sample was measured at 450 nm. Finally, the levels of BNP, NT-pro BNP, Mb, and cTnI were quantified by the standard curve and expressed in ng/L.

#### 2.2.5 Examination of pathological changes

In a 0 °C environment, the heart tissue was divided into three equal parts after clipping off the excess arterial and venous blood vessels. The apex of the heart, which is the lowest part, contains only the left ventricle. The middle part includes the left ventricle, right ventricle, and interventricular septum. The uppermost part comprises the left atrium, right atrium, and interatrial septum. The apex of the hearts of each group of rats was immersed in 4% paraformaldehyde for subsequent pathological evaluation. The remaining two parts were quickly stored at −80 °C. The middle part of the heart was used for transcriptome and arachidonic acid-targeted metabolomic studies. The apex of the hearts of each group of rats was stained with hematoxylin–eosin (H&E) to obtain sections for pathological assessment. Morphological and pathological observation and analysis were performed under a microscope to judge the structural effects of PPT on the heart tissue.

### 2.3 Transcriptome analysis for PPT-induced cardiotoxicity

#### 2.3.1 RNA extraction, library construction, and sequencing

Heart tissue cells from eight rats each in the CON and PPT groups were selected for transcriptomic analysis. Total RNA was isolated from myocardial cells using the TRNzol Universal RNA Sample Preparation Kit (Invitrogen, United States) following the manufacturer’s instructions. Nanodrop 2000 (NanoDrop, Wilmington, DE, United States) was used to detect the concentration and purity of the extracted RNA. RNA integrity was detected by agarose gel electrophoresis, and the RIN value was determined on an Agilent 2100 system (Agilent Technologies, CA, United States). The total amount of RNA required was ≥1 μg for a single database establishment. Sequencing libraries were constructed using Illumina Truseq™ RNA library Prep kit (Illumina, Nebraska, United States) following the manufacturer’s protocol.

#### 2.3.2 Data processing and enrichment analysis

Differential expression analysis was conducted using DESeq2 software (1.20.0). Significant differentially expressed genes (DEGs) between groups were determined using the following criteria: *p* ≤ 0.05 and |log_2_ Fold Change| ≥ 1. Functional enrichment analysis, including Gene Ontology (GO) and Kyoto Encyclopedia of Genes and Genomes (KEGG), were performed to identify significant GO terms and enriched metabolic pathways related to the DEGs (*p* ≤ 0.05) compared with the whole-transcriptomic background. GO functional enrichment and KEGG pathway analysis were conducted using the clusterProfiler package (3.4.4).

### 2.4 Arachidonic acid metabolism-based targeted metabolomics study on cardiotoxicity induced by PPT

#### 2.4.1 Sample pretreatment

Heart samples from rats in the CON (*n* = 8) and PPT (*n* = 8) groups were randomly selected and thawed at 4 °C before metabolomic analysis. Each tissue sample (50 mg) was mixed with a ten-fold amount of the BHT protein precipitant and 10 μL of the internal standard at a concentration of 1 μg/mL, mixed for 20 s, and centrifuged at 4 °C and 14000 rcf for 10 min for protein precipitation. Next, the supernatant (400 μL) was purified using an Oasis HLB 96-well plate. The eluent was dried under nitrogen gas and subjected to UPLC-Q-TOF/HRMS analysis for metabolomics evaluation. An equal amount (10 μL) from each sample was mixed with the QC sample to monitor the repeatability and stability of the instrument.

#### 2.4.2 UPLC-QTRAP-MS analysis

Chromatographic separation was achieved using an Agilent 1290 Infinity LC system (Agilent, United States) with a Waters ACQUITY UPLC BEH C18 (1.7 μm, 2.1 × 50 mm) chromatographic column at a maintained temperature of 35 °C. The tissue sample injection volume was set to 2 μL, and the temperature of the autosampler was fixed at 4 °C. The gradient mobile phase was a mixture of 0.1% formic acid in water (phase A) and 0.1% formic acid in acetonitrile (phase B), which was pumped at a flow rate of 0.4 mL/min. The optimal elution procedure of phase B was set as follows: 0–1 min, 30%; 1–9 min, 30%–90%; 9–11 min, 90%; 11–11.1 min, 90%–20%; 11.1–14 min, 20%. Mass spectrometry data detection and acquisition was performed on the QTRAP mass spectrometry (5500 QTRAP, SCIEX, United States, ESI) instrument in the MRM mode. The ion pair information of all 15 arachidonic acid metabolites is shown in Supplementary Table S2. Mass spectra were obtained in the negative ion mode. Mass spectrometry parameters were set as follows: source temperature, 500°C; Ion Source Gas1 (Gas1), 50; Ion Source Gas2 (Gas2), 50; Curtain gas (CUR), 30; Ion Spray Voltage Floating (ISVF), −4500 V.

#### 2.4.3 Methodological study

Ion flow chromatography (XIC) is used to extract a standard sample of metabolites to determine whether each can be quantitatively analyzed by mass spectrometry. Six QC samples were injected continuously for analysis, and RSD values of peak area and retention time were calculated to assess the stability and repeatability of the experiment. The linear range of 15 metabolites was determined by a linear test.

#### 2.4.4 Data processing

The chromatographic peak area and retention time were determined using Multiquant 3.0.2 software (SCIEX, United States). Metabolite identification and absolute quantification were performed using the standard substance of the 15 arachidonic acid metabolites to correct retention time. Differential metabolites with fold change >1.5 or <0.67 with *p* < 0.05 were selected as candidates. R (version 4.0.3) and R packages were used for all multivariate data analyses and modeling. Data were mean-centered using Pareto scaling. Models were built on orthogonal partial least-squares discriminant analysis (OPLS-DA) and partial least-squares discriminant analysis (PLS-DA). All the models evaluated were tested for overfitting using permutation test methods.

### 2.5 Statistical analysis

Descriptive statistics were used to characterize the groups studied. The mean and standard deviation, medians (interquartile range IQR), and number (percentage) were used to depict normally distributed data, non-normally distributed continuous data, and categorical data, respectively. According to the application’s requirements, the toxicity observation scale data were compared using Pearson’s chi-square test. Other recorded data were compared using Student’s t-test for categorical variables or the Mann–Whitney *U* test for quantitative variables. IBM SPSS Statistics for Windows (version 25.0, IBM Corp; Armony, NY) was used to analyze all data. All statistical tests were two-tailed, and *p <* 0.05 was considered statistically significant.

## 3 Results

### 3.1 Evaluation of cardiotoxicity induced by PPT (IPE and AOE)

#### 3.1.1 Acute cardiotoxicity induced by PPT

**FIGURE 1:**
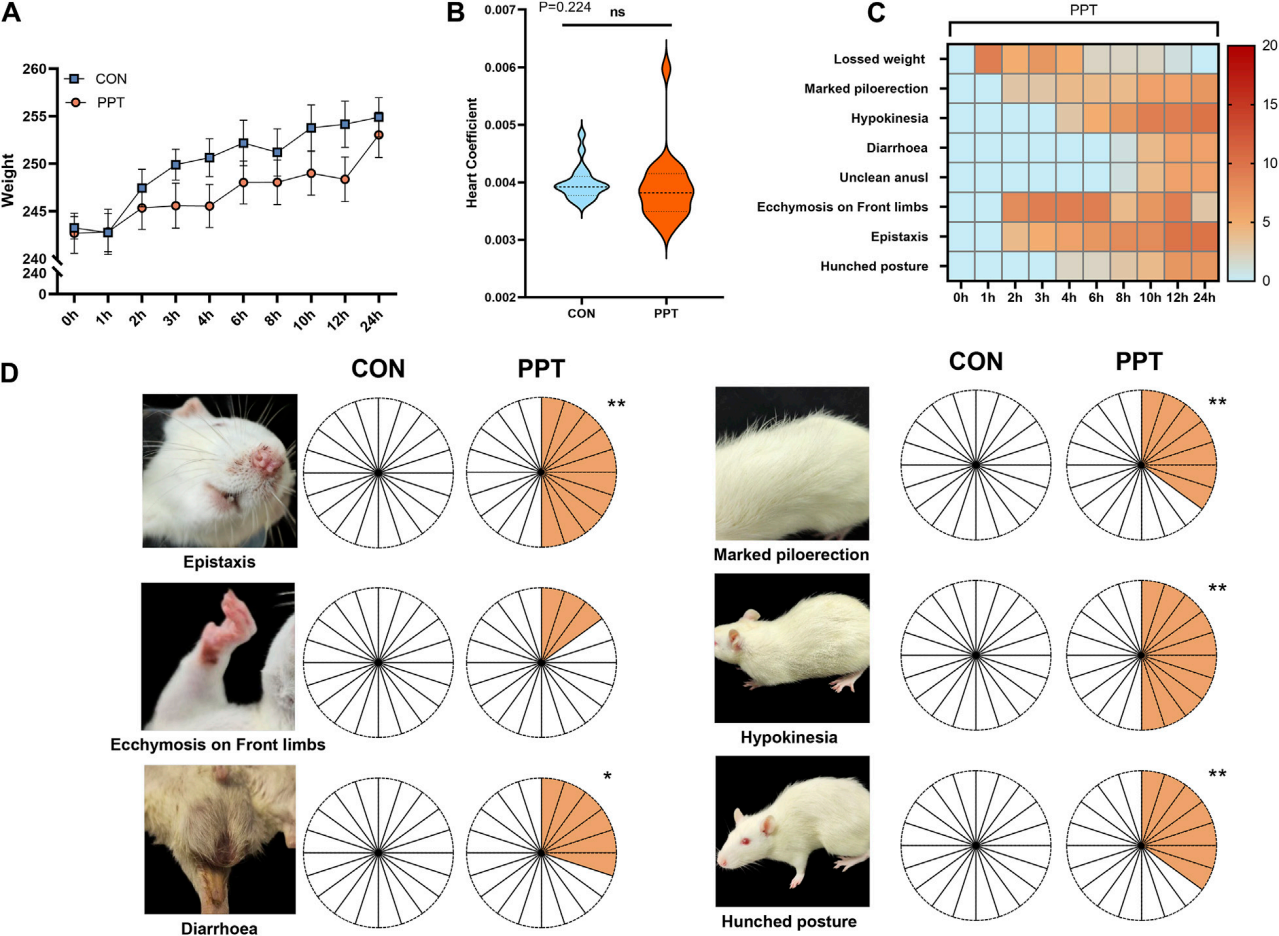
Variations of weight, organ index of heart, and objective phenotypes of male SD rats (IPE). **(A)** Weight comparison between groups. **(B)** Comparison of the organ index of the heart in each group. **(C)** Changes in behavior performances in the PPT group within 24 h. **(D)** Behavior performance comparison between groups. Values are presented as mean ± SD and median ± IQR. *p < 0.05, **p < 0.01 vs. the CON group.

Statistical analysis of rat weight showed that the weight of animals in the CON and PPT groups significantly increased after administration (*p* < 0.05). Compared with the CON group, the weight difference in rats in the PPT group before and after 24 h of administration was not statistically significant (*p* > 0.05) (Figure 1A. The statistical analysis of the organ index of the heart showed no statistically significant difference between the PPT and CON groups (*p* > 0.05) (Figure 1B and Supplementary Table S3). Behavior observation showed no significant changes in the CON group. Compared with the CON group, rats in the PPT group exhibited varying degrees of upright and rough hair, lazy movement, unclean anus, and oronasal hemorrhage. An obvious ecchymosis on the feet (*p* < 0.01) after 2 h of administration, obvious symptoms of oronasal hemorrhage (*p* < 0.05) after 3 h of administration, lazy movement (*p* < 0.05) after 6 h of administration, symptoms of upright and rough hair (*p* < 0.05) after 10 h of administration, and arched back (*p* < 0.01) after 12 h of administration (Figures 1C, D and Supplementary Table S1) were noted in rats of the PPT group.

#### 3.1.2 Serum myocardial zymogram indicators

**FIGURE 2:**
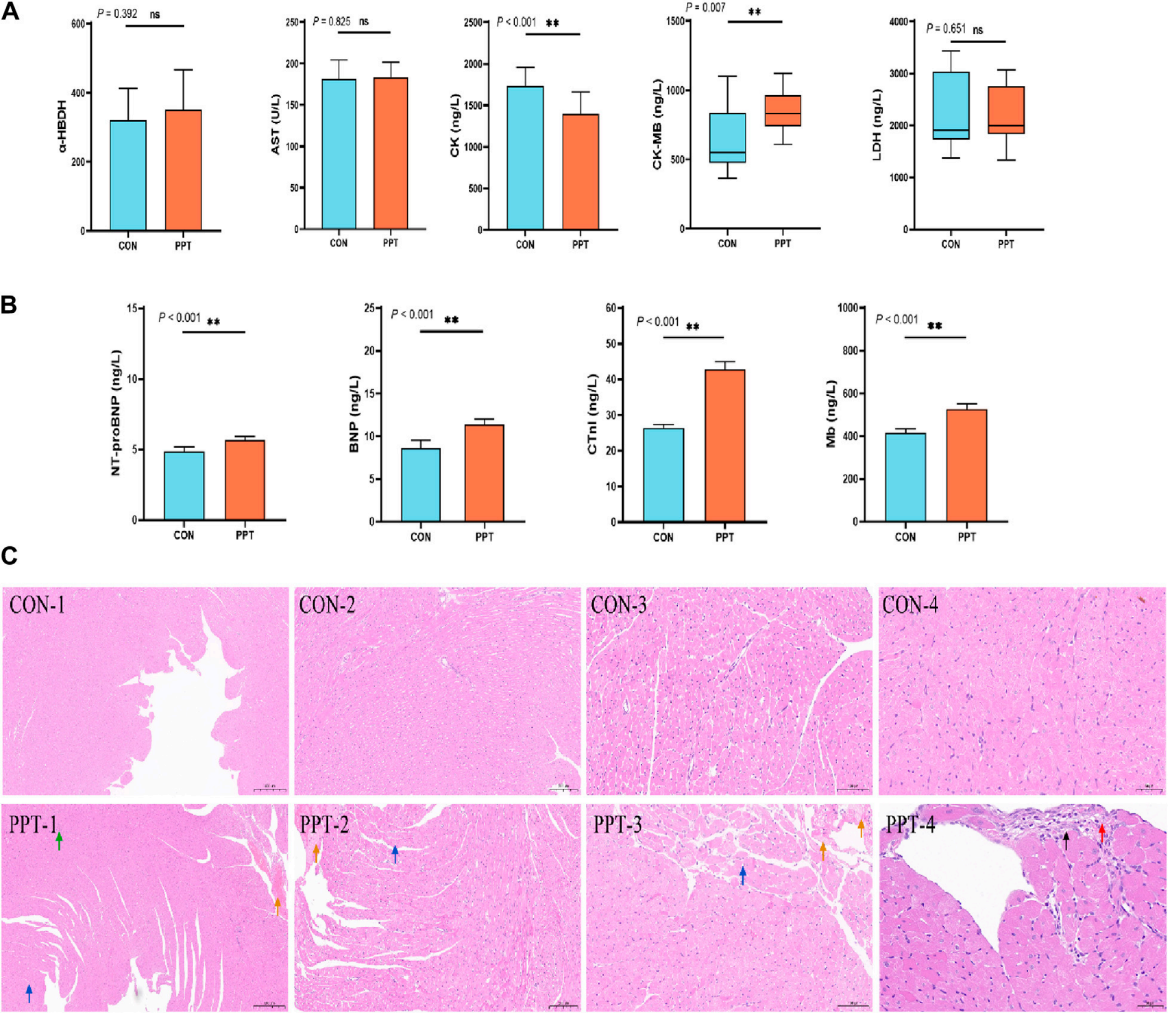
Cardiac toxicity induced by PPT in male SD rats (AOE). **(A)** Serum myocardial zymogram indicators. **(B)** Cardiac injury markers. **(C)** Pathological sections of heart tissues in each group. Green arrow: blood vessel congestion; yellow arrow: insoluble fibrin; blue arrow: loose cytoplasm of myocardial cells in the left ventricular wall, with visible vacuoles in the cytoplasm; black arrow: connective tissue hyperplasia; red arrow: inflammatory infiltration. Values are presented as mean ± SD and median ± IQR. *p < 0.05; **p < 0.01 vs. the CON group.

CK, CK-MB, LDH, AST, and α-HBD, commonly used indicators reflecting the degree of myocardial cell damage (Xuan and Jian, 2016), were assessed to evaluate the degree of cardiac damage caused by PPT. The results of serum biochemical parameters are shown in Figure 2A. Compared with the CON group, the levels of CK in the serum of rats in the PPT group were significantly reduced (*p* < 0.01), while those of CK-MB were significantly increased (*p* < 0.01); the levels of LDH, AST, and α-HBD showed an upward trend but without statistically significant differences (*p* > 0.05). These results indicated that after 24 h of gavage of the PPT aqueous solution to rats, serum myocardial enzyme concentrations fluctuated, with the most significant increase in CK-MB and decrease in CK levels.

#### 3.1.3 Cardiac injury markers

BNP, cTnI, Mb, and NT-proBNP levels are mainly used to diagnose symptoms of heart failure and myocardial infarction in clinical medicine (Kang et al., 2017). We evaluated cardiac injury by detecting the concentrations of BNP, cTnI, Mb, and NT-proBNP in the serum. ELISA results showed that compared with the CON group, the levels of BNP, NT-proBNP, cTnI, and Mb in the serum of rats in the PPT group were significantly increased (*p* < 0.05), indicating that the heart tissue of rats was damaged after 24 h of administering the PPT aqueous solution (Figure 2B).

#### 3.1.4 Pathological manifestations

Pathological manifestations of the rat hearts were observed under an optical microscope. Histopathological results showed that the heart tissue’s endocardial and epicardia structures were clearly visible, the vascular structure was intact, the myocardial fibers were tightly arranged, the cell boundaries were clearly visible, and the cell morphology was consistent with no expansion, atrophy, degeneration, necrosis, or inflammatory cell infiltration in the CON group. Compared with the CON group, the PPT group showed a certain degree of cardiac damage, mainly manifested as vascular congestion, focal connective tissue hyperplasia, inflammatory infiltration, cytoplasmic looseness of myocardial cells, and visible vacuoles in the cytoplasm. According to the International Harmonization of Nomenclature and Diagnostic Criteria for Lesions in Rats and Mice (INHAND) proposal (www.goReni.org.), the cardiac tissue of rats in the CON group was scored grade 0 and considered normal. The pathological rating of heart tissue in the PPT group was grade 1, and the pathological changes exceeded the normal range. Therefore, administration of the PPT aqueous solution caused some damage to the heart. The specific details are shown in Figure 2C. The pathological scoring criteria are shown in Supplementary Table S4.

### 3.2 Transcriptome analysis

**FIGURE 3:**
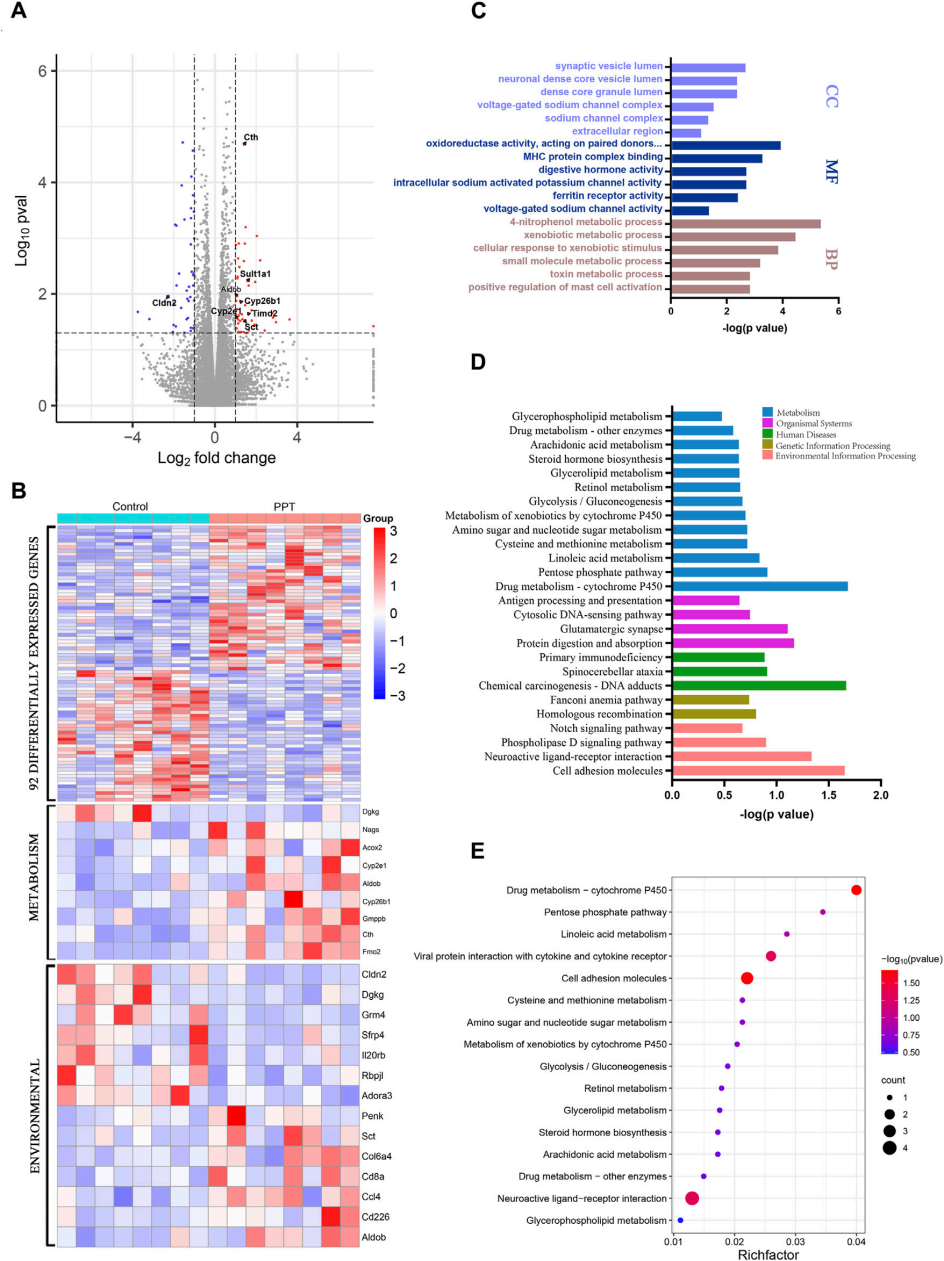
Transcriptome analysis of cardiotoxicity induced by PPT in male SD rats (TEE1). **(A)** Volcano plot for different groups. **(B)** Clustering heatmap of DEGs. **(C)** GO enrichment analysis for DEGs. **(D)** KEGG enrichment analysis for DEGs. **(E)** KEGG enrichment analysis for DEGs and bubble plots.

A total of 92 DEGs, including 50 upregulated and 42 downregulated genes, were identified between the CON and PPT groups (Figure 3A). Cluster analysis of DEGs divided the CON group and PPT groups distinctly (Figure 3B). To determine the main biological functions related to DEGs, GO enrichment analysis was conducted (Figure 3C). Exposure to 120 mg/kg PPT primarily induced alterations in acute inflammatory responses, toxin metabolism, exogenous metabolism, positive regulation of mast cell activation, and immune response regulatory signaling pathways. In terms of biological processes (BP), DEGs were mainly enriched in 4-nitrophenol metabolism, toxin metabolism, xenobiotic metabolic process, and positive regulation of mast cell activation. In terms of molecular functions (MF), DEGs were mainly enriched in oxidoreductase activity, MHC protein complex binding, intracellular sodium-activated potassium channel activity, and acetyl CoA activity. In terms of cellular components (CC), DEGs were mainly enriched in the synaptic vesicle lumen, voltage-gated sodium channel complex, dense core granule lumen, and sodium channel complex. Notably, to determine the potential enriched pathways related to DEGs, KEGG pathway enrichment analysis was performed for the 92 DEGs, with the results shown in Figures 3D, E. The primary altered metabolic or signaling pathways included arachidonic acid metabolism, linoleic acid metabolism, glycolysis, pentose phosphate pathway, amino acid metabolism, phospholipase D signaling pathway, and the Notch signaling pathway.

### 3.3 Arachidonic acid metabolism disorder due to PPT-induced cardiotoxicity

#### 3.3.1 Methodological investigation

The experimental methodology is shown in Supplementary Figure S1 and Supplementary Table S2, with the method repeatability and the sample stability meeting all requirements for metabolomics analysis (RSD (peak area) < 30%). The linear equations for all metabolites reached *r* > 0.999, and the chromatographic separation of each metabolite was good, with sharp and symmetrical peak shapes.

#### 3.3.2 Metabolic profiling

**FIGURE 4:**
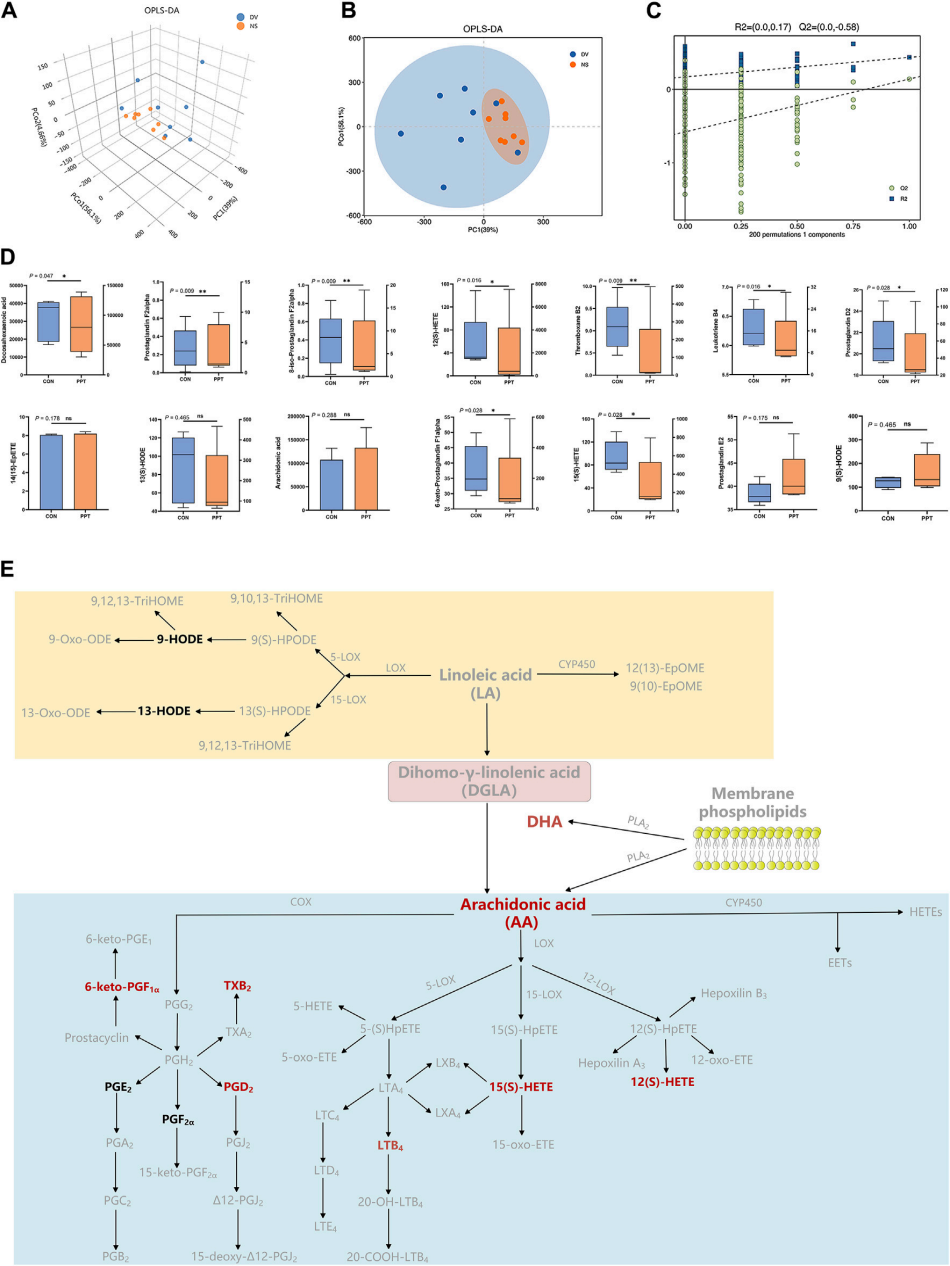
Targeted metabolomics analysis of PPT-induced cardiotoxicity in male SD rats (TEE2). **(A)** OPLS-DA 3D score plots in ESI^−^ mode. **(B)** OPLS-DA score plots in ESI^−^ mode. **(C)** Permutation test for OPLS-DA in ESI^−^ mode. **(D)** Quantitative results of arachidonic acid metabolites in different groups. **(E)** Differential metabolites of arachidonic acid metabolic pathways; potential metabolites in red and other metabolites in black. Data presented as mean ± SD and median ± IQR. ^*^ *p* < 0.05, ^**^ *p* < 0.01 vs. the CON group.

Quantitative analysis was conducted on the 15 metabolites of the arachidonic acid pathway; except for leukotriene D4, which was not detected below the detection limit, the levels of the remaining 14 metabolites were measured in the PPT and CON groups (Table 2; Figure 4D). To distinguish the metabolic profiles among the CON and PPT groups, OPLS-DA in ESI^−^ mode (negative ion mode) was performed for cluster analysis. Significant separation of the CON and PPT groups in OPLS-DA confirmed the reliability and reproducibility of the test method (Figures 4A, B). Furthermore, the permutation plot of PLS-DA showed that the model had no overfitting and was reliable (Figure 4C). Eight differential metabolites with *p* < 0.05 and FC > 1.5 were selected as differential metabolites, including 12(S)-HETE, 8-iso-PGF_2α_, TXB2, 6-keto-PGF_1α_, 15(S)-HETE, DHA, PGD_2,_ and LTB_4_. Compared with the CON group, all eight metabolites were upregulated after administration of PPT (Figures 4D, E).

**TABLE 2:** Quantitative detection results of arachidonic acid targeted metabolomics ( $\bar{x}$ ± SD, M (IQR)).

Component name	Fold change	*p*	PPT	CON
Docosahexaenoic acid	2.520	0.020	68103.368 ± 42758.492	27026.253 ± 10888.772
Prostaglandin F2alpha	2.580	0.272	1.337 (4.25)	0.431 (0.99)
8-iso-Prostaglandin F2alpha	2.800	0.328	107.961 (696.87)	61.201 (108.27)
12(S)-HETE	3.549	0.407	12.127 (10.16)	9.095 (1.62)
Thromboxane B2	3.538	0.407	6.779 (3.04)	6.339 (0.72)
Leukotriene B4	1.321	0.456	23.499 (10.33)	20.821 (4.79)
Prostaglandin D2	1.337	0.461	8.195 (0.31)	8.12 (0.43)
14 (15)-EpETE	0.989	0.528	8.140 ± 0.251	8.230 ± 0.304
13(S)-HODE	0.789	0.691	108099.432 (62053.14)	105866.389 (34865.62)
Arachidonic acid	1.059	0.734	110328.950 ± 45779.398	104170.482 ± 20686.505
6-keto-Prostaglandin F1alpha	1.280	0.773	129.055 (183.59)	111.987 (57.94)
15(S)-HETE	1.149	0.822	38.822 (2.67)	38.566 (4.2)
Prostaglandin E2	0.986	0.840	103.414 (111.9)	133.963 (66.07)
9(S)-HODE	0.959	0.862	126.553 ± 78.072	132.019 ± 39.093

### 3.4 Integrated analysis of genes, metabolites, myocardial enzymes, and cardiac injury markers

**FIGURE 5:**
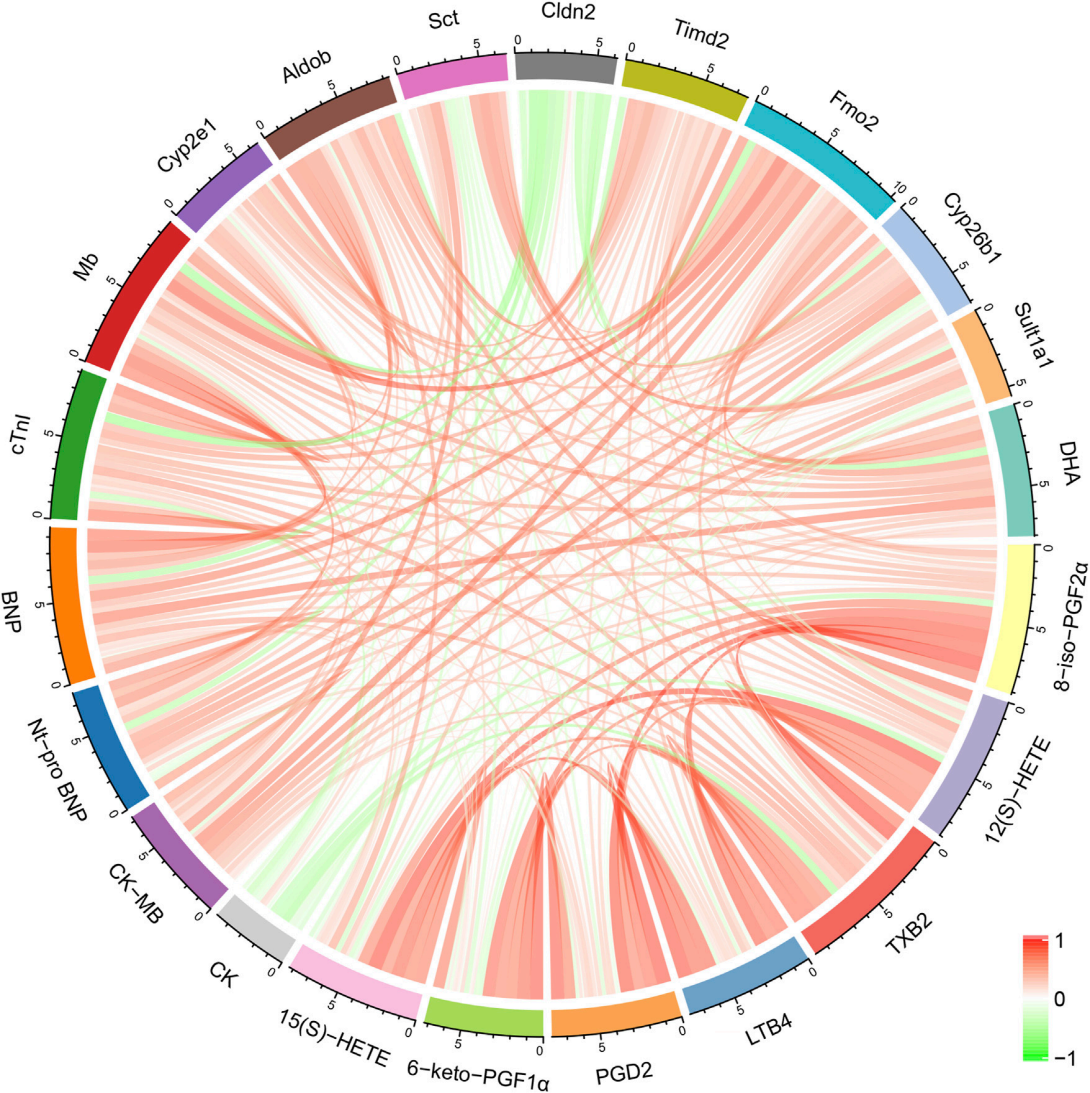
Correlation analysis between differential metabolites, major differentially expressed genes, myocardial enzymes, and cardiac injury markers.

Correlation analysis was performed to assess the inter-relationships among genes, metabolites, myocardial enzymes, and cardiac injury markers. As shown in Figure 5, Cyp2e1 was positively correlated with several differential metabolites, including 8-iso-PGF2α, 12(S)-HETE, TXB2, LTB4, PGD2, and 6-keto-PGF1α. Aldob was positively correlated with 8-iso-PGF2α, 12(S)-HETE, TXB2, and PGD2. DHA, a differential metabolite, was positively correlated with differential genes Fmo2 and Timd2, as well as heart injury markers BNP and Mb.

## 4 Discussion

### 4.1 Toxicological research on cardiotoxicity induced by PPT (IPE and AOE)

In our previous work, we demonstrated that administering 1.2 g/kg/day of DV to SD rats induced toxicity (Liu et al., 2020). For the clinical context, the commonly prescribed dosage range for DV is 3–9 g for a 60-kg adult human, equivalent to an experimental dose of approximately 0.6 g/kg/day for rats when adjusted for the body surface area (Xu et al., 2002). Given that the PPT content in DV is 91 mg/g (Wang et al., 2017), the experimental dose for this study was calculated as 109 mg kg–1 (1.2 × 91 = 109.2 mg kg–1), which is roughly double the clinically relevant dose. Therefore, we selected 120 mg/kg to robustly evaluate the potential cardiotoxicity effects of PPT. This study revealed for the first time that rats in the PPT group exhibited symptoms of diarrhea, marked piloerection, epistaxis (nosebleeds), hunched posture, and hypokinesia (reduced movement), which intensified over time within 24 h after administration of 120 mg/kg PPT. Interestingly, we report for the first time that the symptoms of ecchymosis (bruising) in the PPT group rats decreased within 24 h after administration. Although the body weight of the PPT group rats was significantly lower than that of the CON group between 2 and 12 h after administration, there was no significant difference in body weight between the two groups by 24 h. We speculate that the above situation may be because the rats may have triggered a self-repair or regulatory process in the first 24 h of exposure to PPT, especially between 13 h and 24 h. In this study, the body weight of the rats increased significantly within 24 h, and even that in the CON group rats increased by nearly 10 g within 24 h. This may be because we used 7-week-old SD rats in a rapid growth stage of sexual maturity which were made to fast, and water access was prevented for 8 h before administration. The rats were made to fast, and water access was prevented 1 h after administration. After providing sufficient food and water 1 h after administration, the body weight of the rats increased significantly within 24 h.

There was a statistically significant decrease in CK content (*p* < 0.05) and a remarkable increase in CK-MB content (*p* < 0.05) in the PPT group compared to the CON group. This pattern of change suggests possible myocardial injury in the experimental rats, as CK-MB is a more specific marker of cardiac muscle damage than CK. This unequal expression suggests that SD rats may be in the early stage of myocardial injury, as CK-MB is mainly present in cardiac muscle cells. Its elevation is usually more sensitive than CK—when myocardial injury is in the early stage, only a small portion of B subunits are released into peripheral blood due to the presence of M subunits in myocardial cells, and CK produced by other cells in the body is absorbed by surplus CK-MB, resulting in a decrease in CK levels (Bertinchant et al., 2000; Ma et al., 2023). In clinical settings, the increase in BNP levels is the specific gold standard for diagnosing cardiac dysfunction. BNP is synthesized and secreted by ventricular muscle cells, and its level can increase 200–300 times when myocardial infarction—cardiac overload—or dilation occurs (McGrath et al., 2005; Sun et al., 2023). CTnI and Mb mainly exist in the myocardium and can be detected in the serum 2 h after cardiac damage (Apple, 1999; Kyriazidis et al., 2023). These characteristics enable BNP, cTnI, and Mb to serve as more sensitive indicators of cardiac damage. These results indicate that PPT may cause a certain degree of damage to the rats’ hearts. To further evaluate the cardiotoxicity due to PPT, we conducted pathological observations on the heart tissues of rats after administering PPT intragastrically. The PPT group showed a certain degree of cardiac damage. According to the degree of pathological changes in heart tissue, INHAND was used to stratify the histopathological results into five grades: 0, 1, 2, 3, and 4. As the grade ascends, the corresponding degree of tissue damage progressively intensifies. Grade 0 signifies that the tissue alterations remain within normal limits, whereas grade 1 indicates that the tissue changes surpassed the normal range. Grade 4, on the other hand, denotes that the lesions have encompassed the entire tissue or organs. In this study, the pathological assessment of heart tissue from rats in the PPT group was classified as grade 1, suggesting that PPT induced notable morphological alterations in the rat heart tissue. In summary, administering 120 mg/kg PPT within 24 h may cause cardiac injury.

### 4.2 Mechanism underlying acute cardiac toxicity induced by PPT in rats

In this study, transcriptomics was employed to characterize the gene expression in rats following PPT administration. PPT disrupted the genetic equilibrium in rats, resulting in significant alterations in 92 genes, with 50 genes being upregulated and 42 downregulated. GO analysis showed that these genes were mainly involved in biological processes, such as oxidoreductase activity, toxin metabolism, 4-nitrophenol metabolic process, and synaptic vesicle lumen. Pathway enrichment analysis for DEGs revealed a significant involvement in arachidonic acid metabolism, linoleic acid metabolism, glycolysis, pentose phosphate pathway, amino acid metabolism, phospholipase D signaling pathway, and the Notch signaling pathway. Arachidonic acid metabolism, linoleic acid metabolism, phospholipase D signaling pathway, and Notch signaling pathway are closely related to inflammation. Glycolysis and pentose phosphate pathways are involved in the energy metabolism of the heart. Therefore, PPT may lead to cardiac energy metabolism disorders and inflammation by altering related genes/proteins. To verify this hypothesis, targeted metabolomic technology was used in this study to characterize the arachidonic acid metabolic profile involved in the inflammatory response. Among the 14 detected metabolites, eight differential metabolites were upregulated, including: DHA, AA, and their metabolite; LTB4, PGD2, and 8-iso-PGF2α; TXB2, 12(S)-HETE, and 15(S)-HETE. The main differential metabolites are closely related to the occurrence, development, and resolution of inflammation, which play a crucial role in the development and progression of cardiovascular disease. LTB4, the pro-inflammatory lipid mediator produced by the metabolism of LOXs (Lipoxygenases), increases vascular permeability by activating the CysLT2 receptor in blood vessels, mediating myocardial ischemia and reperfusion injury (Jiang et al., 2008). 12-HETE is produced primarily by the 12-LOX, which exerts a chemotaxis effect that promotes the exudation, adhesion, and aggregation of leukocytes. 12-HETE contributes to sustained inflammation and increased cardiomyocyte death, elevating pro-inflammatory markers such as MCP-1 and IL-6 and resulting in monocyte and neutrophil recruitment during the acute phase post-MI (Kuzuya et al., 1987; Shibata et al., 1988). 12-HETE is involved in oxidative stress. It promotes ROS generation, which activates the transcription factor NF-κB and regulates the expression of many genes. 12 HETE significantly increases the levels of phosphorylated NF-κB, which in turn leads to β cell dysfunction (Weaver et al., 2012). Inflammatory reactions and massive PG levels are induced in the heart tissues after ischemic conditions (Kong et al., 2016; Tang et al., 2017) or DOX treatment (Robison and Giri, 1987). Similarly, in our study, the level of PGD2, a COX-derived AA metabolite, in the PPT rats was remarkably higher than that in the CON group. Cardiac-generated prostanoids, particularly PGD2, directly mediate cardiac myocyte apoptosis after myocardial ischemia (Qiu et al., 2012).

Through integrated analysis, we observed that Cyp2e1 was positively correlated with a series of differential metabolites, including 8-iso-PGF2α, 12(S)-HETE, TXB2, LTB4, PGD2, and 6-keto-PGF1α. Cyp2e1 (cytochrome P450 2E1) overexpression can increase the necrosis and apoptosis of cardiomyocytes (Zhang et al., 2011). Cyp2e1 can promote injury in diabetic heart microvascular endothelial cells by increasing the production of ROS under high glucose conditions, and quercetin can reduce the damage caused by high sugar-induced cardiac microvascular endothelial cells by inhibiting Cyp2e1 (Chen et al., 2018). The expression of Cyp2e1 increases in alcoholic cardiomyopathy, which mediates the transformation of cardiac fibroblasts into myofibroblasts, leading to myocardial fibrosis (Zhang et al., 2023). Aldob is significantly positively correlated with 8-iso-PGF2α, 12(S)-HETE, TXB2, and PGD2. Fructose-bisphosphate aldolase B (Aldob) is a tetrameric glycolytic enzyme mainly involved in synthesizing D-glyceraldehyde 3-phosphate and glycerone phosphate from D-glucose as part of the glycolysis and carbohydrate degradation pathways. As one of the indicators of the CS4P score, ALDOB is not cardiac-specific but reflects multiorgan dysfunction, systemic inflammation, and immune activation (Iborra-Egea et al., 2020a; Iborra-Egea et al., 2020b). DHA has anti-inflammatory effects, producing anti-inflammatory and inflammation-resolving mediators such as resolvins, protectins, and maresins (omega-3 fatty acids and inflammatory processes from molecules to man). DHA was positively correlated with the differential gene Fmo2 (flavin-containing monooxygenase 2), as well as the heart injury markers BNP and Mb. The expression of FMO2 in the heart is primarily enriched in fibroblasts rather than myocytes. Fmo2 can prevent cardiac fibrosis. A significant downregulation of FMO2 is consistently observed in hearts after myocardial infarction (MI) in rodents. In this study, after exposure to PPT, both anti-inflammatory DHA and fibrosis-inhibiting Fmo2 were upregulated, suggesting that the heart is fighting against the interference of PPT to maintain homeostasis.

Collectively, the mechanism of PPT-induced acute cardiotoxicity involves oxidative stress, apoptosis, inflammatory response, and disorder of the energy metabolism.

### 4.3 Limitations

The objective of this study was to confirm acute cardiotoxicity induced by PPT in male SD rats within 24 h and to investigate the underlying mechanisms. However, due to euthanasia and sampling of all experimental animals 24 h post-administration, continuous observation was not feasible, making it impossible to ascertain the reversibility of the observed IPE phenotype. The dosage employed in this study was based on clinical application and prior studies, with a single dose administered without exploring the dose–response relationship. Future studies should encompass a range of doses to gain a more comprehensive understanding. Furthermore, while this study utilized transcriptomics to detect expressional changes across the entire transcriptome, the lack of quantitative analysis (qRT-PCR) precluded in-depth expression intensity analysis. Moreover, functional validation has not been performed on the identified key genes or differential metabolites. As such, future research should aim to silence critical genes at the cellular level using RNAi technology or inhibit them with corresponding inhibitors. Similarly, inhibitors or gene-knockout technology can be employed at the whole animal level to verify their impact on the onset and progression of PPT-induced cardiac toxicity. By manipulating the levels of differential metabolites, toxicological alterations in animals can be observed and potential biomarkers and therapeutic drugs can be uncovered.

## 5 Conclusion

**FIGURE 6:**
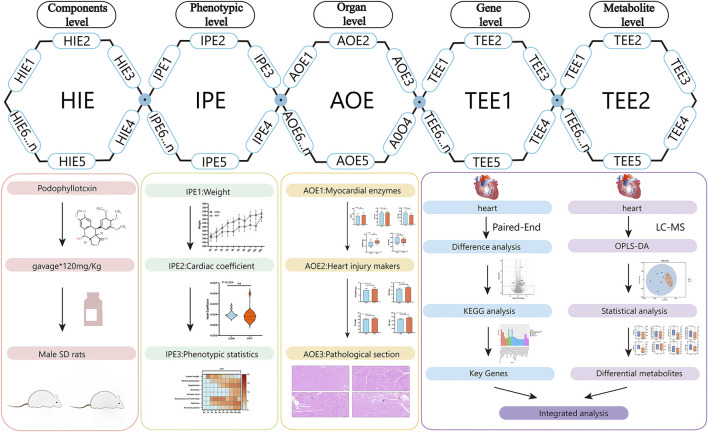
Full-text summary diagram.

The results of this study provide a preliminary understanding of the molecular mechanisms of cardiotoxicity caused by PPT. Injury phenotypic changes were assessed based on alterations in body weight, behavioral changes (IPE). Adverse outcomes evidence was evaluated through elevations in myocardial enzymes and cardiac injury biomarkers, along with histopathological observations (AOE). Furthermore, the study illustrated transcriptome characteristics and arachidonic acid metabolism profiles (TEE). Finally, the mechanism of acute cardiotoxicity caused by PPT involves oxidative stress, apoptosis, inflammatory response, and disordered energy metabolism. The developed TEC concept can serve as a useful tool to integrate data from multi-platform systems biology techniques, with the potential of broad-ranging application to other herbal medicines to determine new indicators of organ injury and toxicant studies to analyze omics data and identify the mechanisms underlying toxicity (Figure 6).

## Figures and Tables

**Table:** 

**12(S)-HETE**	12(S)-Hydroxy eicosatetraenoic acid
**13(S)-ODE**	13(S)-hydroxy octadecadienoic acid
**14(15)-EpETE**	14,15-Epoxyeicosatetraenoic acid
**15(S)-HETE**	15(S)-Hydroxy eicosatetraenoic acid
**6-keto-PGF**_**1α**_	6-keto-Prostaglandin F1alpha
**eight-iso-PGF**_**2α**_	eight-iso-Prostaglandin F2alpha
**9(S)-ODE**	9(S)-hydroxy octadecadienoic acid
**AOE**	Adverse Outcomes Evidence
**ARA**	Arachidonic acid
**AST**	Aspartate aminotransferase
**BNP**	Brain natriuretic peptide/brain natriuretic peptide
**BP**	Biological process
**CC**	Cellular component
**CK**	Creatine kinase
**CK-MB**	Creatine kinase-MB isoenzyme
**CON**	Control
**cTNI**	Cardiac troponin I
**DEGs**	Different expression genes
**DHA**	Docosahexaenoic acid
**DV**	*Dysosma versipellis*
**GO**	Gene Ontology
**H&E**	Hematoxylin–eosin
**HIE**	Harmful ingredients evidence
**IPE**	Injury phenotype evidence
**IQR**	Inter quartile range
**KEGG**	Kyoto Encyclopedia of Genes and Genomes
**LDH**	Lactate dehydrogenase
**LTB₄**	Leukotriene B4
**LTD₄**	Leukotriene D4
**MF**	Molecular functional
**MYO/MB**	myoglobin
**NT-proBNP**	N-terminal pro-brain natriuretic peptide
**PGD₂**	Prostaglandin D2
**PGE₂**	Prostaglandin E2
**PGF**_**2α**_	Prostaglandin F2alpha
**PPT**	Podophyllotoxin
**TEC**	Toxicological evidence chain
**TEE**	Toxic events evidence
**TXB₂**	Thromboxane B2
**XIC**	Extracting ion flow chromatography
**α-HBD**	Alpha-hydroxybutyrate dehydrogenase.

## Data Availability

All raw reads data were stored in the NCBI sequence read archive (SRA accession number: PRJNA1154551).
