# Supplementary material for: Potential mechanisms underlying podophyllotoxin-induced cardiotoxicity in male rats: toxicological evidence chain (TEC) concept
Source: Front Pharmacol. 2024 Sep 24;15:1378758. doi: 10.3389/fphar.2024.1378758 (PMC11463157; doi:10.3389/fphar.2024.1378758)
Supplement: Supplementary file 1 [file DataSheet1.PDF]

Supplementary Material

Contents:

Table S1 Animal toxicity behavior observation scale

Table S2 MRM and standard curve information of 15 arachidonic acid pathway metabolites

Table S3 Heart weight scale of rats in each group

Table S4 The grading systems for pathological tissue changes#

Table S1. Animal toxicity behavior observation scale

Observation Time	Toxicity Behavior	Animal Proportion		
		PPT	CON	Chi-Square Test
6h	Lossed weight	4/20	0/20	-
	Marked piloerection	4/20	0/20	-
	Hypokinesia	5/20	0/20	P = 0.047
	Unclean anusl	0/20	0/20	-
	Diarrhoea	0/20	0/20	-
	Ecchymosis on Front limbs	9/20	0/20	P = 0.000
	Epistaxis	7/20	0/20	P = 0.003
	Erythema on the dorsal surface	0/20	0/20	-
	Hunched posture	2/20	0/20	-
	Death	0/20	0/20	-
12h	Lossed weight	12/20	0/20	P = 0.000
	Marked piloerection	6/20	0/20	P = 0.008
	Hypokinesia	9/20	0/20	P = 0.000
	Unclean anusl	6/20	0/20	P = 0.008
	Diarrhoea	1/20	0/20	-
	Ecchymosis on Front limbs	9/20	0/20	P = 0.000
	Epistaxis	10/20	0/20	P = 0.000
	Erythema on the dorsal surface	0/20	0/20	-
	Hunched posture	7/20	0/20	P = 0.003
	Death	0/20	0/20	-
24h	Lossed weight	2/20	0/20	-
	Marked piloerection	7/20	0/20	P = 0.003
	Hypokinesia	10/20	0/20	P = 0.000
	Unclean anusl	6/20	0/20	P = 0.008
	Diarrhoea	1/20	0/20	-
	Ecchymosis on Front limbs	3/20	0/20	-
	Epistaxis	10/20	0/20	P = 0.000
	Erythema on the dorsal surface	0/20	0/20	-
	Hunched posture	7/20	0/20	P = 0.003
	Death	0/20	0/20	-

Table S2. MRM and standard curve information of 15 arachidonic acid pathway metabolites

Metabolite Name	QC RSD	Mass Info	Retention Time (min)	Linear	R	Linear range (ng/ml)	LOD (ng/ml)	LOQ (ng/ml)	ULOQ (ng/ml)
Arachidonic acid	0.008	303.2 / 259.2	7.442	y = 0.01489 x + 0.13985	r = 0.99944	0.25-1000	0.05	0.25	1000
Docosahexaenoic acid	0.038	327.1 / 229.2	7.287	y = 0.00419 x + 0.05541	r = 0.99925	0.25-1000	0.05	0.25	1000
12(S)-HETE	0.017	319.1 / 179.0	5.547	y = 0.01233 x -0.06198	r = 0.99996	0.25-1000	0.05	0.25	1000
15(S)-HETE	0.037	319.1 / 219.0	5.299	y = 0.00253 x -0.00917	r = 0.99991	0.25-1000	0.05	0.25	1000
14(15)-EpETE	0.012	317.1 / 207.0	5.882	y = 0.00308 x -0.01138	r = 0.99785	0.25-1000	0.05	0.25	1000
9(S)-HODE	0.026	295.1 / 171.1	5.193	y = 0.00685 x -0.07859	r = 0.99984	0.25-1000	0.05	0.25	1000
13(S)-HODE	0.038	295.0 / 195.0	5.151	y = 0.01037 x + 0.16279	r = 0.99931	0.25-1000	0.05	0.25	1000
Leukotriene B4	0.014	335.2 / 195.0	3.871	y = 0.01051 x -0.02960	r = 0.99987	0.25-1000	0.05	0.25	1000
Leukotriene D4		495.3 / 177.2		y = 0.00911 x -0.10834	r = 0.99958	0.25-1000	0.05	0.25	1000
6-keto-Prostaglandin F1α	0.010	369.3 / 163.0	0.884	y = 0.01741 x -0.11266	r = 0.99972	0.25-1000	0.05	0.25	1000
8-iso-Prostaglandin F2α	0.008	353.1 / 309.3	2.092	y = 0.02847 x + 0.01161	r = 0.99968	0.25-1000	0.05	0.25	1000
Prostaglandin D2	0.014	351.2 / 271.3	2.273	y = 0.03877 x -0.35361	r = 0.99885	0.25-1000	0.05	0.25	1000
Prostaglandin E2	0.005	351.2 / 271.3	2.258	y = 0.04250 x -0.79358	r = 0.99944	0.25-1000	0.05	0.25	1000
Prostaglandin F2α	0.008	353.1 / 309.3	2.091	y = 0.03219 x + 0.01030	r = 0.99975	0.25-1000	0.05	0.25	1000
Thromboxane B2	0.017	369.2 / 169.0	1.759	y = 0.00935 x -0.03738	r = 0.99984	0.25-1000	0.05	0.25	1000

Table S3. Heart weight scale of rats in each group

Sample	heart weight（g）	
	**PPT**	**CON**
1	1.127	1.17
2	1.031	1.07
3	0.915	1.047
4	0.852	1.009
5	0.839	0.951
6	0.917	1.077
7	1.029	0.953
8	0.858	1.208
9	0.927	0.987
10	0.956	1.032
11	0.908	1.029
12	1.092	1.038
13	1.098	0.942
14	0.996	0.916
15	0.906	1.023
16	0.919	0.968
17	1.455	0.899
18	1.012	0.97
19	0.875	1.028
20	0.952	0.992

Table S4. The grading systems for pathological tissue changes[#]

	PPT		CON	
	**Number**	**Level**	**Number**	**Level**
blood vessel congestion	5/8	1	2/8	1
insoluble fibrin	4/8	1	0/8	0
the cytoplasm of myocardial cells in the left ventricular wall is loose, with visible vacuoles in the cytoplasm	6/8	1	1/8	0
connective tissue hyperplasia	5/8	1	0/8	0
inflammatory infiltration	4/8	1	0/8	0
Comprehensive pathologic grading of heart tissue	1		0	

[#]The grading was performed according to The International Harmonization of Nomenclature and Diagnostic Criteria for Lesions in Rats and Mice (INHAND) (www.goReni.org.). Level 0 indicates that under research conditions, considering factors such as the animal's age, gender, and strain, the tissue is considered normal. However, changes observed under other conditions may be deemed abnormal. Level 1 indicates that pathological changes just exceed the normal range. Level 2 indicates that pathological changes can be observed, but are not yet severe. Level 3 indicates that the pathological changes are evident and likely to worsen. Level 4 indicates that the pathological changes are very severe, with the lesion occupying the entire tissue or organ.
